# Combining Community Wastewater Genomic Surveillance with State Clinical Surveillance: A Framework for SARS-CoV-2 Public Health Practice

**DOI:** 10.1007/s12560-022-09531-2

**Published:** 2022-08-18

**Authors:** Ted Smith, Rochelle H. Holm, Ray Yeager, Joseph B. Moore, Eric C. Rouchka, Kevin J. Sokoloski, Erin M. Elliott, Daymond Talley, Vaneet Arora, Sarah Moyer, Aruni Bhatnagar

**Affiliations:** 1 Christina Lee Brown Envirome Institute, School of Medicine, University of Louisville, 302 E. Muhammad Ali Blvd., Louisville, KY 40202 USA; 2 Center for Integrative Environmental Health Sciences, School of Medicine, University of Louisville, 500 S. Preston St., Louisville, KY 40202 USA; 3 Diabetes and Obesity Center, School of Medicine, University of Louisville, 580 S. Preston St., Louisville, KY 40202 USA; 4 Department of Biochemistry and Molecular Genetics, School of Medicine, University of Louisville, 319 Abraham Flexner Way, Louisville, KY 40202 USA; 5 Department of Microbiology and Immunology, School of Medicine, University of Louisville, 505 S. Hancock St., Louisville, KY 40202 USA; 6 Center for Predictive Medicine for Biodefense and Emerging Infectious Diseases, University of Louisville, 505 S. Hancock St., Louisville, KY 40202 USA; 7 Morris Forman Water Quality Treatment Center, Louisville/Jefferson County Metropolitan Sewer District, 4522 Algonquin Parkway, Louisville, KY 40211 USA; 8 Division of Laboratory Services, Kentucky Department for Public Health, 100 Sower Blvd., Suite 204, Frankfort, KY 40601 USA; 9 Department of Pathology and Laboratory Medicine, University of Kentucky, 800 Rose St., Lexington, KY 40536 USA; 10 Department of Health Management and System Sciences, School of Public Health and Information Sciences, University of Louisville, 485 E. Gray St., Louisville, KY 40202 USA; 11 Department of Public Health and Wellness, Louisville-Jefferson County Metro Government, 400 E. Gray St., Louisville, KY 40202 USA

**Keywords:** Community health, COVID-19, Local government, Sewer, Variant, Wastewater-based epidemiology

## Abstract

This study aimed to develop a framework for combining community wastewater surveillance with state clinical surveillance for the confirmation of severe acute respiratory syndrome coronavirus 2 (SARS-CoV-2) variants within the community and to provide recommendations on how to expand on such research and apply the findings in public health responses. Wastewater samples were collected weekly from 17 geographically resolved locations in Louisville/Jefferson County, Kentucky (USA), from February 10 to December 13, 2021. Genomic surveillance and quantitative reverse transcription PCR (RT-qPCR) platforms were used to screen for SARS-CoV-2 in wastewater, and state clinical surveillance was used for confirmation. The study results highlighted an increased epidemiological value of combining community wastewater genomic surveillance and RT-qPCR with conventional case-auditing methods. The spatial scale and temporal frequency of wastewater sampling provided promising sensitivity and specificity for gaining public health screening insights about SARS-CoV-2 emergence, seeding, and spread in communities. Improved national surveillance systems are needed against future pathogens and variants, and wastewater-based genomic surveillance exhibits great potential when coupled with clinical testing. This paper presents evidence that complementary wastewater and clinical testing are cost-effectively enhanced when used in combination, as they provide a strong tool for a joint public health framework. Future pathogens of interest may be examined in either a targeted fashion or using a more global approach where all pathogens are monitored. This study has also provided novel insights developed from evidence-based public health practices.

**Supplementary Information:**

The online version contains supplementary material available at 10.1007/s12560-022-09531-2.

## Introduction

Many communities across the United States and worldwide use quantitative reverse transcription polymerase chain reaction (RT-qPCR) platforms to quantify severe acute respiratory syndrome coronavirus 2 (SARS-CoV-2) variants. This is largely because of the low cost and effort involved as well as the real-time availability of the associated data. However, this approach carries several disadvantages. Most notably, once a variant of concern is identified, there is often a lag time in the availability of primers and probes, PCR cannot always distinguish between variants, and there is loss of historical data about a wide range of mutations that may have been useful in post-hoc analyses (Boudet et al., 2021; Yaniv et al., 2021). Community wastewater samples can be used to further identify the diversity of SARS-CoV-2 variants on a geographic scale (Crits-Christoph et al., 2021; Fontenele et al., 2021; Izquierdo-Lara et al., 2021; Jahn et al., 2021; Nemudryi et al., 2020). As one of the communities in the United States employing next-generation sequencing (NGS) and comprehensive bioinformatic analysis of wastewater samples, weekly for 10 months, during the coronavirus disease 2019 (COVID-19) pandemic, Louisville /Jefferson County, Kentucky (USA), is uniquely positioned to provide insights and recommendations for a coupled approach to the sequencing of clinical and wastewater samples with geographic resolution. To further leverage the public health response value of this approach, genomic surveillance was applied at seventeen wastewater collection sites in the county and underserved urban communities were intentionally oversampled (Yeager et al., 2021). Policy recommendations can be provided for other regions trying to implement genomic surveillance from these evidence-based practices. The aim of this study was to present a framework for combining community wastewater genomic surveillance with traditional clinical surveillance that confirms SARS-CoV-2 variants within the community. Further, the study aimed to expand this research field and extend its applications to public health response.

## Methods

### Wastewater Sample Collection and Handling

Wastewater was collected as 24-h time-weighted composite samples from seventeen sites in Louisville/Jefferson County, Kentucky (USA), from February 10 to December 13, 2021. The samples were collected weekly. The sites are a combination of street lines and pump stations, which represent neighborhood or zip-code level areas, and treatment centers covering larger geographic areas (Yeager et al., 2021). Samples were transported on ice to the University of Louisville for RT-qPCR analysis and processed using the methodologies previously described (Rouchka et al., 2021).

### Genomic Surveillance

On a weekly basis, one sample per site was sent for sequencing, regardless of the Ct value, and a total of 697 samples were sequenced using NGS. To achieve high sensitivity, the samples were enriched for SARS-CoV-2 nucleic acids using the Swift Biosciences SNAP protocol for SARS-CoV-2. Samples were barcoded using the Swift Biosciences indexing kit and sequenced on an Illumina NextSeq 500 using the NextSeq Mid Output Kit v.5 for 300 cycles (2 × 150 bp reads). Low-quality sequences were trimmed using Trimmomatic (Bolger et al., 2014) to avoid false-negative single nucleotide polymorphism (SNP) detection. The sequences were directly aligned with the SARS-CoV-2 reference genome assembly (NC_045512.2) using BWA (Li, 2013). The reads were then trimmed using a Swift Biosciences primer clip (Addetia et al., 2020) to remove the primer read regions to avoid variant calling within these regions. BCFTools mpileup (Li et al., 2009) was used to summarize the coverage of mapped reads and detected variants. Variants occurring at a frequency ≥ 0.05 with at least five sequences confirming the variant were filtered for further analysis. The pattern of mutations within each sample was compared and classified into the variants of concern (VOC), variants of interest (VOI), and variants under monitoring (VUM), as cataloged by outbreak.info. A mean sequencing depth of five was required across the mutations associated with a particular variant. The per-base coverage and overall viral coverage varied from week to week and from site to site (Fig. S1), and was largely dependent on viral concentration, as determined by the Ct values (Fig. S2). Wastewater samples with at least 75% of mutations associated with a specific variant were marked as being likely to contain the particular variant. The results of this analysis were available within a week of the sample being collected and were then shared with county and state public health officials.

### Ethics

The University of Louisville Institutional Review Board classified this project as Non-Human Subject Research (reference #: 717950).

## Results and Discussion

Since May 2020, the University of Louisville (UofL) has pioneered the application of wastewater-based epidemiology (WBE) as a partner to both Louisville Metro Public Health and Wellness (LMPHW) and the Kentucky Department of Public Health (DPH) in Louisville/Jefferson County. The initial focus was on quantifying the virus recovered in the community and correction facility wastewater to help inform public health officials about the intensity and spread of the COVID-19 disease. However, by February 2021, this work at the UofL expanded to include full genomic sequencing of recovered viral RNA fragments, which allowed for the provision of estimates of the likelihood of variants of SARS-CoV-2 being present in community wastewater catchment areas across the Louisville/Jefferson County, Kentucky area. The work included the Centers for Disease Control and Prevention (CDC) variants of concern (VOC), variants of interest (VOI), and some variants of attention but with no classification. The initial surveillance that started in February 2021 included: B.1.1.7, B.1.351, B.1.427, B.1.429, P.1, R.1, B.1.618, B.1.623, B.1.617.1, B.1.617.2, B.1.617.3, and B.1.621. C.37 was added on July 5, 2021. On July 26, 2021, B.1.525, B.1.526, and P.2 were added. The Omicron variant (B. 1.1.529) was added on November 30, 2021. Genomic sequencing results were typically available 4–5 days after wastewater sample collection. Sample collection was conducted on Mondays, RNA extraction and QA/QC on Tuesdays, sample library preparation on Wednesdays, sequence loading on Thursdays, and bioinformatics analysis on Friday evenings or Saturday mornings, depending on the sequence completion time. During the initial months of genomic analysis of the wastewater sampling, there were several opportunities to explore the utility of this type of sampling method at the community scale. As a result, a loose combination between wastewater analysis efforts and the state’s clinical genomic surveillance emerged. Collaboration between state public health and academic centers of excellence (formal or informal) presents an excellent opportunity for improved situational awareness and shared learning in the public health response practice. Initiation of these collaborations often only involved reaching out to their counterparts in the academic or state public health center and sharing ideas and needs. This initial simple exchange of ideas between UofL, LMPHW, and DPH developed into a program that produced the successful scenarios presented here.

Four scenarios demonstrate the manner in which complementary data from each activity were discovered. First, because community wastewater sampling is a pooled test that effectively samples a population of > 7000 using toilets connected to the wastewater system, the wastewater sample is more likely to detect an emerging variant than would through sparse sequencing of individual clinical nasopharyngeal swabs. This was demonstrated in early April 2021, when wastewater genomic surveillance identified the Gamma (P.1) SARS-CoV-2 variant, and UofL communicated this to the county and state for a geographically and demographically targeted public health response. The variant was confirmed one week later when its first clinical case within the same wastewater catchment area was identified using clinical sequencing. Since then, there have been numerous comparisons between the catchment areas and clinical trends with this same “wastewater community screening and clinical confirmation” variant approach. This observation is similar to that of Jahn et al. (2021), who reported their detection of B.1.1.7 in Switzerland wastewater treatment samples prior to detection by clinical sampling.

The second scenario occurred in late April 2021 when the DPH sequenced clinical samples from a Kentucky Senior Living Center outbreak reported to be caused by the R.1 variant. The DPH requested verification with the UofL wastewater database to determine whether this particular variant had been detected anywhere in the county, so that the risk of spread could be better understood, while the United States Government SARS-CoV-2 Interagency Group classified the variant (Cavanaugh et al., 2021). Fortunately, the weekly data showed no evidence that R.1 had spread to Louisville/Jefferson County.

In the third instance, the B.1.526 variant was detected at one of the wastewater collection sites on August 16, 2021, a time at which B.1.617.2 (Delta) was dominant across all other locations. Subsequent clinical sequencing yielded a B.1.526 sample collected on September 7, 2021, from an adjacent zip code (Supplementary Fig. S3). This illustrates the early detection capabilities of wastewater surveillance even at low levels and when other variants are circulating.

The fourth instance was the rise of the Omicron variant. South Africa notified the World Health Organization (WHO) of the Omicron variant on November 24, 2021, and it was subsequently added to the UofL bioinformatics analysis as a target for both future and retrospective wastewater analyses. On December 17, 2021, the Omicron variant was detected in a wastewater sample that was collected on December 13, 2021. On December 21, 2021, the Omicron variant was detected in a clinical sample collected from the county during the preceding week. By the time it was identified in the wastewater, the Omicron variant had already infected members in the community, but the delays in clinical sequencing meant that the wastewater efforts and framework were able to alert county officials four days earlier than clinical detection.

Since February 2021, weekly updates from the UofL academic partners have been provided to the LMPHW, thereby graphically representing the presence of wastewater variants within a rolling 4-week period (Supplement Fig. S4). Variants tend to appear or disappear on a weekly basis, and a moving 4-week period allowed stability in the observation of variants. Moving reporting from a formal technical document to less than a dozen slides allowed rapid understanding even among non-genomic and non-bioinformatic professionals and has been accepted as efficacious data in a deliverable format as received by the LMPHW (see Supplement Fig. S4 for an example reference of report). While other genomic studies have focused on wastewater treatment center samples (Crits-Christoph et al., 2021; Jahn et al., 2021; Nemudryi et al., 2020; Smyth et al., 2021), the benefit of a geographically resolved sampling design is that it allows geographically precise identification of variant emergence and neighborhood spread on a weekly basis in the county. Variants observed in smaller wastewater catchment areas, such as neighborhood street lines, were not uniformly observed downstream of the associated treatment centers.

This study complements other sector-wide advocacies to bridge wastewater researchers and public health responders (McClary-Gutierrez et al., 2021). More importantly, this study leveraged a foundational collaboration that existed prior to the pandemic between the UofL, LMPHW, DPH, and the Louisville/Jefferson County Metropolitan Sewer District. The UofL wastewater genomics and bioinformatics laboratories have been involved in whole-genome metagenomics in other contexts, typically metagenomic studies within individuals. Financially, significant components of the sampling and viral analysis were paid for through other mechanisms, including the American Rescue Plan and related federal public health funds. Additionally, UofL is concurrently funded by the NIH through the Institutional Development Award (IDeA) program, CDC for wastewater sampling and testing, and the Commonwealth of Kentucky for wastewater monitoring of correction facilities. These relationships provided the project with effective communication channels with the state public health commissioner and the CDC National Wastewater Surveillance System (NWSS) leadership, which has enabled translation from academic lab settings to sustainable ongoing deployment of the public health response developed in this framework.

**Fig. 1:**
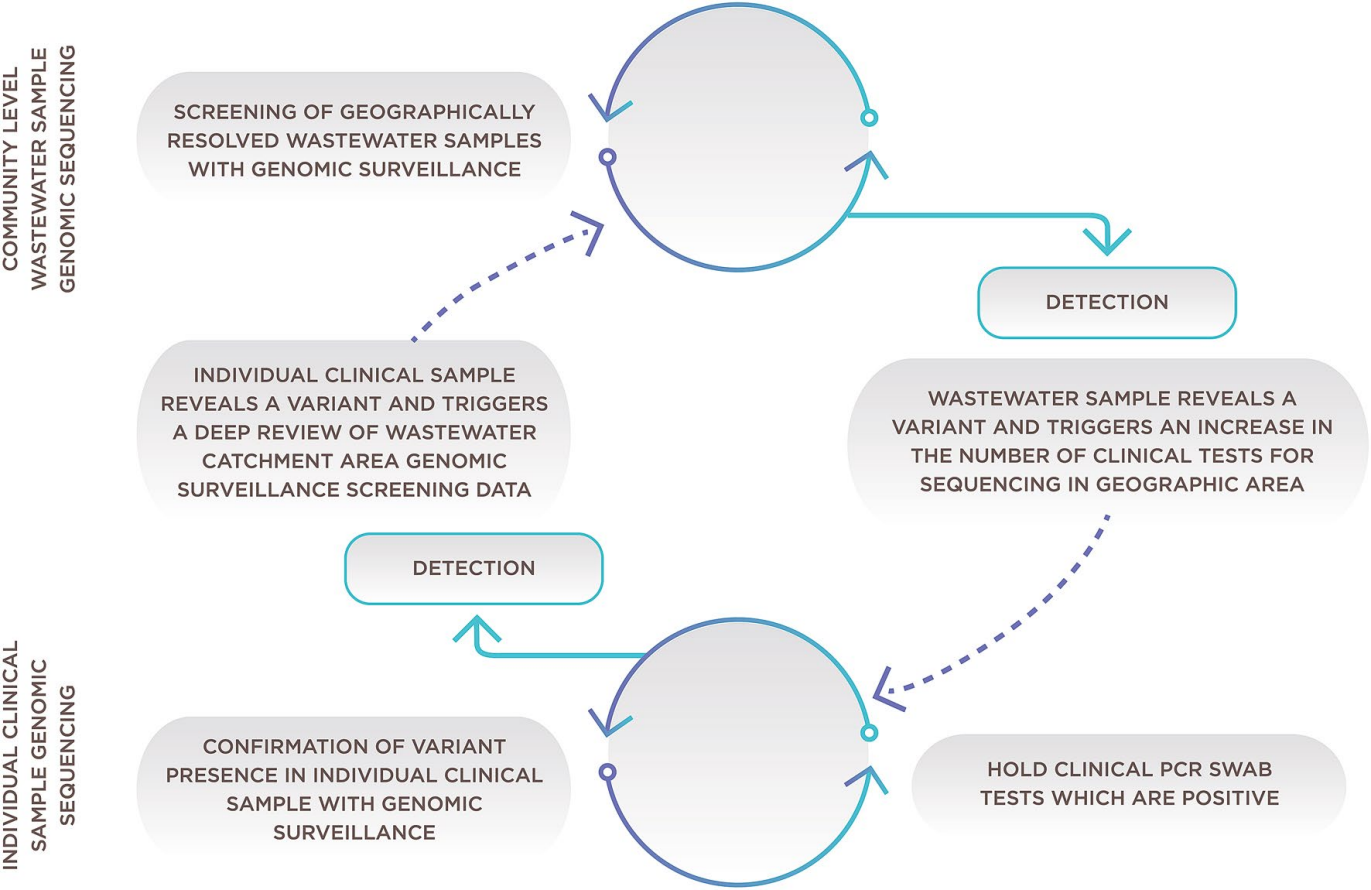
Framework for combining community wastewater genomic surveillance with state clinical surveillance

This work has helped to answer important questions that many communities in the United States and around the world have about the utility of conducting wastewater surveillance as part of genomic surveillance for infectious diseases. Over the course of the pandemic, one of the greatest challenges has been the need to span fields of expertise and capability to reach useful solutions. This framework (Fig. 1) provides valuable insights into the feasibility and value of NGS analysis for SARS-CoV-2 variants in sampling scenarios utilizing smaller community wastewater catchment areas. This work will help public health officials implement improved community sampling schemes, while acknowledging the value of developing coordination with other clinical surveillances. The utility of this approach for COVID-19 certainly extends to many other infectious disease models and other public health hazards, such as toxic exposure.

## Future Policy Recommendations

This research has identified eight key policy recommendations. These include:

1. Determine the spatial scale of wastewater sampling that provides optimal sensitivity and specificity to gain insight into the public health response to variants. For example, it may be less useful to know that a variant is present in a wastewater catchment area covering half of the population than to have neighborhood-scale data. Conversely, there is likely to be a diminishing value in small areas when cost and effort are considered.
2. Define the temporal frequency of community wastewater samples providing an optimal balance of “early warning” and information about seeding and spread. Identifying emerging variants that escape vaccination creates a case for getting ahead of disease spread over time.
3. Analyze the relationship between the spatiotemporal coverage of clinical genomic surveillance and wastewater genomic surveillance. For example, it may be possible to derive a useful level of clinical testing as a function of the wastewater catchment area where the variant was first discovered. Conversely, a clinical case with a new variant first identified at a location could trigger the collection of a series of nested wastewater catchment area samples.
4. Understand the seeding and spread of different variants in the community to improve the understanding of possible entry points in communities and patterns of spread. For example, an initial analysis of historic data in Louisville/Jefferson County suggests neighborhoods near an air logistics hub are more likely to establish new variants over time. It is possible that these data could be connected to a larger database for Kentucky, and perhaps the country, to provide insights into the characteristics of places that could inform new public health interventions.
5. Discern the role of vaccination in suppressing the spread of variants. Wastewater samples may estimate the levels of suppression associated with vaccination coverage in an area to inform transmission rates in communities with high and low vaccination coverage.
6. Determine cost–benefit trade-offs for different configurations of wastewater and clinical genomic surveillance using Louisville/Jefferson County’s historic testing costs.
7. Establish if all variant markers are present but not detectable. All signatures associated with a given variant were rarely detected but the reasons behind this have not been elucidated. Some of this is likely because the bioinformatic alignment is difficult for insertions/deletions (without weakening overall data quality), but for other polymorphisms, it may be because they do not exist.
8. Ascertain increasing throughput without increasing system stress, by asking the question, “How much sequencing depth is needed?”

## Limitations

The RNA sourced from wastewater is of lower quality for the longer sequence reads necessary to identify multiple mutations in a single RNA amplicon. Wastewater detection relies on the capacity of a pathogen to be shed into wastewater, and pathogens or variants with altered fecal shedding may create new challenges for detection. Wastewater surveillance cannot provide data in residential areas that are not served by municipal sewer systems and thus excludes some residences, even in urban areas such as Louisville/Jefferson County. The limit of detection for wastewater surveillance in terms of the smallest number of infections in a community that can be detected is not well established; thus, wastewater surveillance should be used in combination with clinical testing.

## Conclusion

This is an ideal time to improve public health surveillance systems for pathogens, and wastewater-based genomic surveillance represents a feasible and comprehensive complement to established approaches. Furthermore, the addition of genomic sequencing to the wastewater surveillance framework may provide a more comprehensive assessment of regional viral infection surveillance when compared to clinical testing alone. The value of this project to the public health and clinical scientific community rests on the ability to produce compelling evidence that SARS-CoV-2 genomic surveillance is cost-effectively enhanced through a complementary wastewater and clinical testing framework. There is an important value in using a broad perspective approach to identify low-abundance variants early from a few samples. A natural extension of this work is to consider pathogens of interest in wastewater being examined in either a targeted fashion or using a more global approach where all pathogens are monitored. Further, this study has developed novel insights from evidence-based public health practices.

## Supplementary Information

Below is the link to the electronic supplementary material.

Supplementary file1 (DOCX 2075 KB)

Supplementary file2 (XLSX 86 KB)

## Data Availability

All data generated or analyzed during this study are included in this published article and its supplementary information files. Sequencing data is available in NCBI’s Sequence Read Archive under Bioproject PRJNA735936 (https://www.ncbi.nlm.nih.gov/bioproject/PRJNA735936).

## References

[CR1] Addetia A, Lin MJ, Peddu V, Roychoudhury P, Jerome KR, Greninger AL (2020). Sensitive recovery of complete SARS-CoV-2 genomes from clinical samples by use of Swift Biosciences’ SARS-CoV-2 multiplex amplicon sequencing panel. Journal of Clinical Microbiology.

[CR2] Bolger AM, Lohse M, Usadel B (2014). Trimmomatic: A flexible trimmer for Illumina sequence data. Bioinformatics.

[CR3] Boudet A, Stephan R, Bravo S, Sasso M, Lavigne JP (2021). Limitation of screening of different variants of SARS-CoV-2 by RT-PCR. Diagnostics.

[CR4] Cavanaugh AM, Fortier S, Lewis P (2021). COVID-19 outbreak associated with a SARS-CoV-2 R. 1 lineage variant in a skilled nursing facility after vaccination program—Kentucky, March 2021. Morbidity and Mortality Weekly Report.

[CR5] Crits-Christoph A, Kantor RS, Olm MR (2021). Genome sequencing of sewage detects regionally prevalent SARS-CoV-2 variants. Mbio.

[CR6] Fontenele RS, Kraberger S, Hadfield J (2021). High-throughput sequencing of SARS-CoV-2 in wastewater provides insights into circulating variants. Water Research.

[CR7] Izquierdo-Lara R, Elsinga G, Heijnen L (2021). Monitoring SARS-CoV-2 circulation and diversity through community wastewater sequencing, the Netherlands and Belgium. Emerging Infectious Diseases.

[CR8] Jahn, K., Dreifuss, D., Topolsky, I., et al. (2021). Detection and surveillance of SARS-CoV-2 genomic variants in wastewater. *medRxiv*. Retrieved April 28, 2022 from 10.1101/2021.01.08.21249379v2 10.1038/s41564-022-01185-x PMC9352586 35851854

[CR9] Li, H. (2013). Aligning sequence reads, clone sequences and assembly contigs with BWA-MEM. *arXiv preprint, 1303.3997*. Retrieved April 28, 2022 from https://arxiv.org/pdf/1303.3997.pdf

[CR10] Li H, Handsaker B, Wysoker A (2009). The sequence alignment/map format and SAMtools. Bioinformatics.

[CR11] McClary-Gutierrez J, Mattioli M, Marcenac P (2021). SARS-CoV-2 wastewater surveillance for public health action. Emerging Infectious Diseases.

[CR12] Nemudryi A, Nemudraia A, Wiegand T (2020). Temporal detection and phylogenetic assessment of SARS-CoV-2 in municipal wastewater. Cell Reports Medicine.

[CR13] Rouchka EC, Chariker JH, Saurabh K (2021). The rapid assessment of aggregated wastewater samples for genomic surveillance of SARS-CoV-2 on a city-wide scale. Pathogens.

[CR14] Smyth, D. S., Trujillo, M., Cheung, K., et al. (2021). Detection of mutations associated with variants of concern via high throughput sequencing of SARS-CoV-2 isolated from NYC wastewater. *medRxiv*. Retrieved April 28, 2022 from 10.1101/2021.03.21.21253978v1

[CR15] Yaniv K, Ozer E, Shagan M (2021). Direct RT-qPCR assay for SARS-CoV-2 variants of concern (Alpha, B. 1.1.7 and Beta, B. 1.351) detection and quantification in wastewater. Environmental Research.

[CR16] Yeager R, Holm RH, Saurabh K (2021). Wastewater sample site selection to estimate geographically-resolved community prevalence of COVID-19: A sampling protocol perspective. GeoHealth.

