# Supplementary material for: Combining Community Wastewater Genomic Surveillance with State Clinical Surveillance: A Framework for SARS-CoV-2 Public Health Practice
Source: Food Environ Virol. 2022 Aug 18;14(4):410–6. doi: 10.1007/s12560-022-09531-2 (PMC9387882; doi:10.1007/s12560-022-09531-2)
Supplement: Supplementary file 1 — Supplementary file1 (DOCX 2075 KB) [file 12560_2022_9531_MOESM1_ESM.docx]

**Supplementary Materials**

**Combining community wastewater genomic surveillance with state clinical surveillance: a framework for SARS-CoV-2 public health practice**

Ted Smith^a^, Rochelle H. Holm^a*^, Ray Yeager ^a,b^, Joseph B. Moore IV ^a,c^, Eric C. Rouchka^d^, Kevin J. Sokoloski^e,f^, Erin M. Elliott^a,c^, Daymond Talley^g^, Vaneet Arora^h,i^, Sarah Moyer^j,k^, Aruni Bhatnagar^a^

^a^Christina Lee Brown Envirome Institute, School of Medicine, University of Louisville, 302 E. Muhammad Ali Blvd., Louisville, KY 40202, United States

^b^Department of Environmental and Occupational Health Sciences, School of Public Health and Information Sciences, University of Louisville, 485 E. Gray St., Louisville, KY 40202, United States

^c^Diabetes and Obesity Center, School of Medicine, University of Louisville, 580 S. Preston St., Louisville, KY 40202, United States

^d^Department of Biochemistry and Molecular Genetics, School of Medicine, University of Louisville, 319 Abraham Flexner Way, Louisville, KY 40202, United States

^e^Department of Microbiology and Immunology, School of Medicine, University of Louisville, 505 S. Hancock St., Louisville, KY 40202, United States

^f^Center for Predictive Medicine for Biodefense and Emerging Infectious Diseases, University of Louisville, 505 S. Hancock St., Louisville, KY 40202, United States

^g^Louisville/Jefferson County Metropolitan Sewer District, Morris Forman Water Quality Treatment Center, 4522 Algonquin Parkway, Louisville, KY 40211, United States

^h^Kentucky Department for Public Health, Division of Laboratory Services, 100 Sower Blvd., Suite 204, Frankfort, KY 40601, United States

^i^Department of Pathology and Laboratory Medicine, University of Kentucky, 800 Rose St., Lexington, KY 40536, United States

^j^Department of Health Management and System Sciences, School of Public Health and Information Sciences, University of Louisville, 485 E. Gray St., Louisville, KY 40202, United States

^k^Department of Public Health and Wellness, Louisville-Jefferson County Metro Government, 400 E. Gray St., Louisville, KY 40202, United States

*Corresponding author: Rochelle H. Holm (rochelle.holm@louisville.edu)


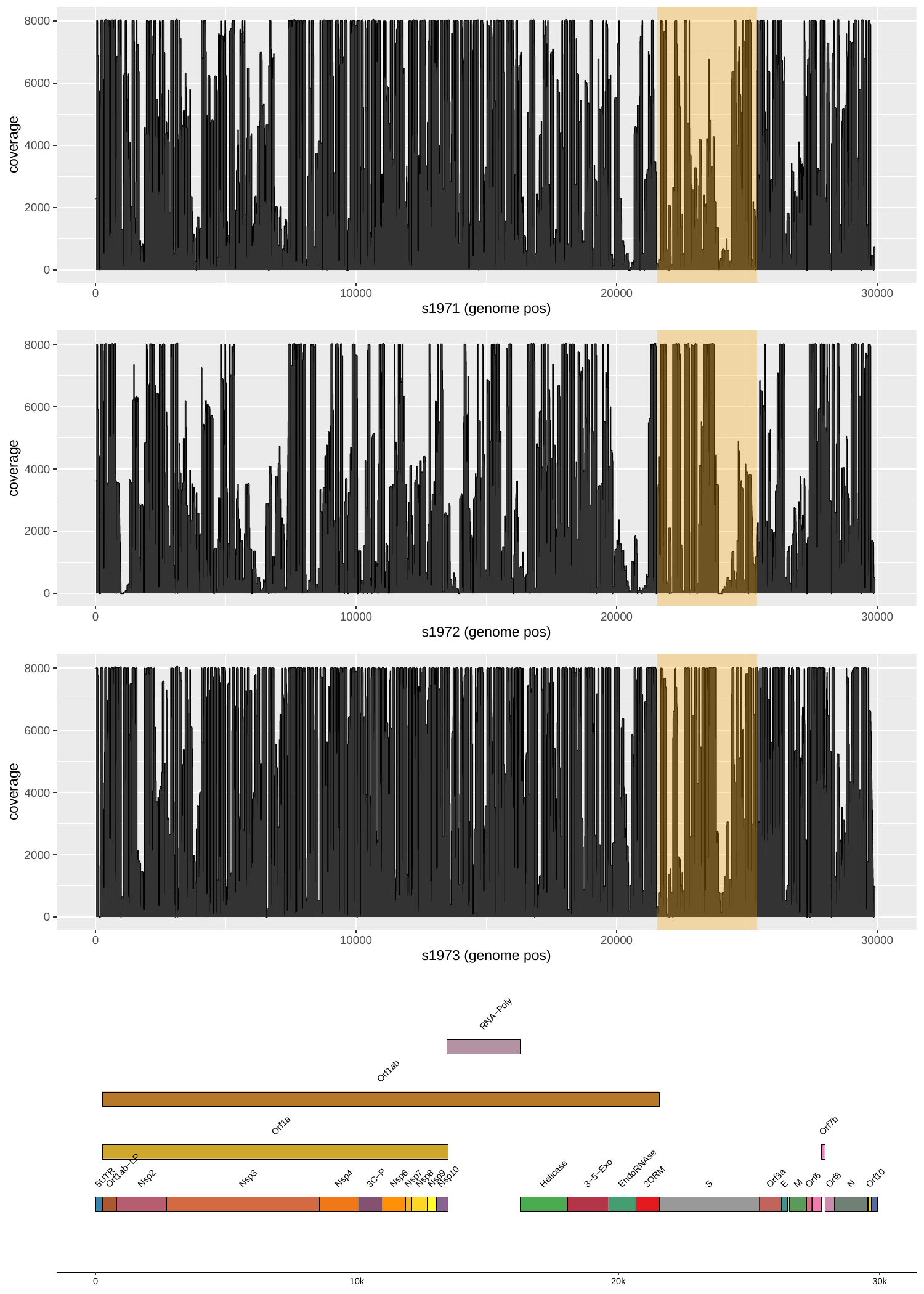


Fig S1 Typical depth coverage (y-axis) across the complete genome (x-axis) at various sampling locations


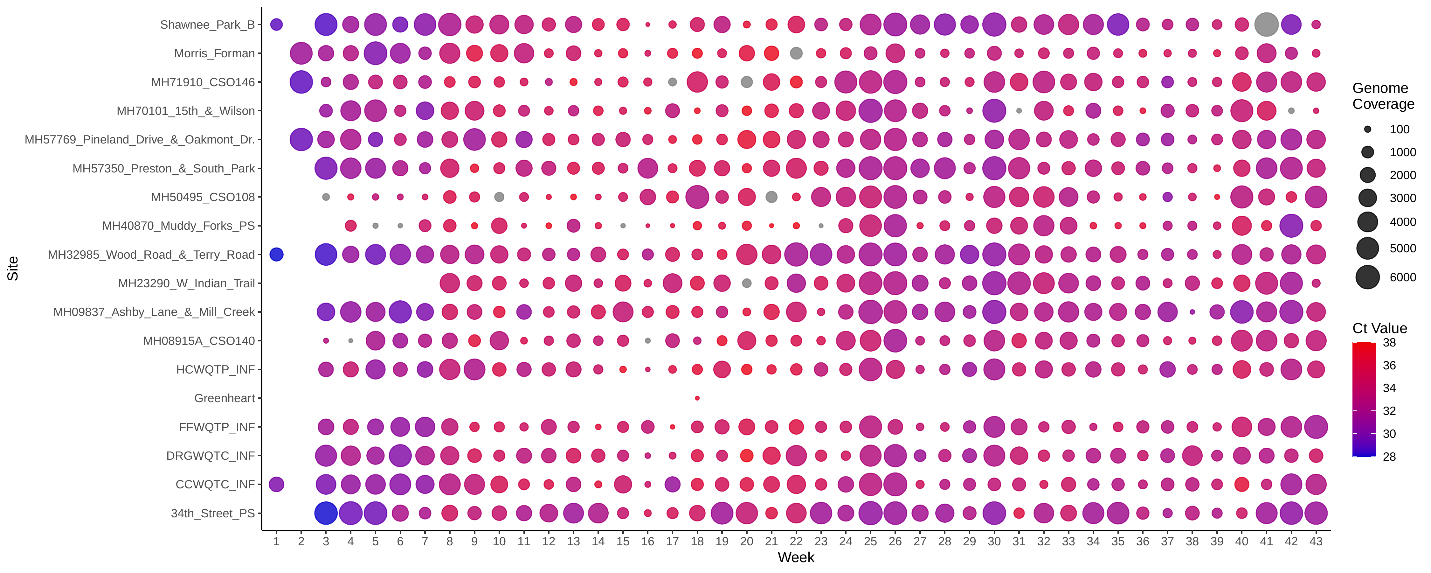


Fig S2 Relationship between genome coverage (area) vs. Ct value (color) for sampled sites


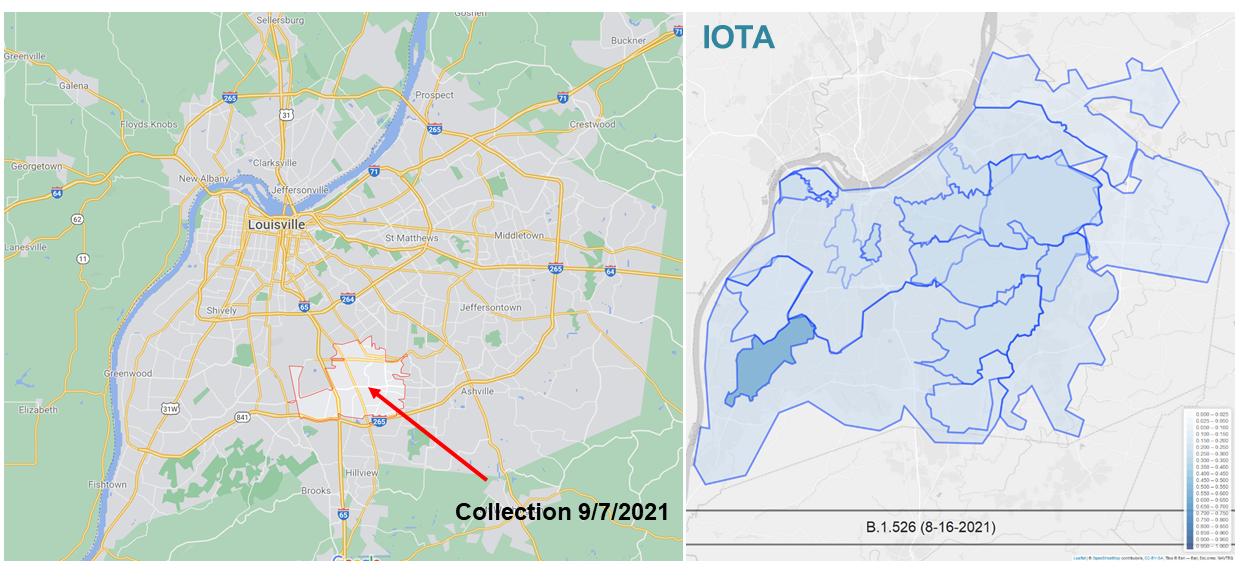


Fig S3 Clinical sequencing yielded a B.1.526 sample from an individual in the area within 40219 zip code (left) from a sample analyzed on September 7, 2021, in Louisville/Jefferson County, Kentucky (USA). Results indicated the presence of B.1.526 in a nearby wastewater collection site two weeks prior, on August 16, 2021 (right).


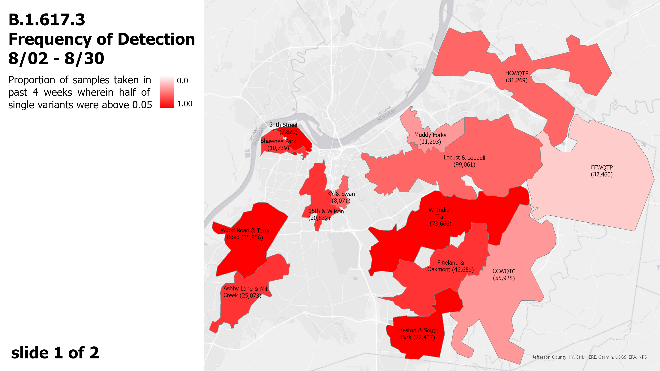

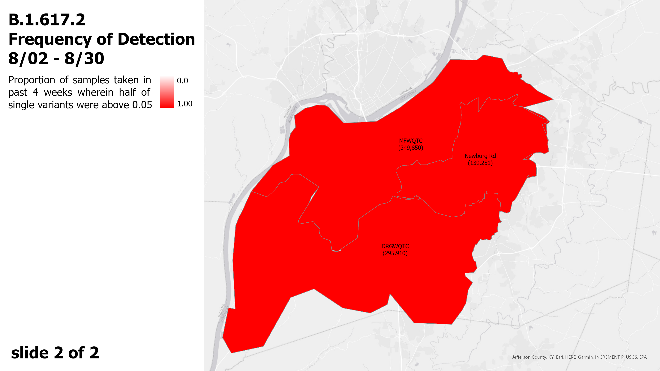


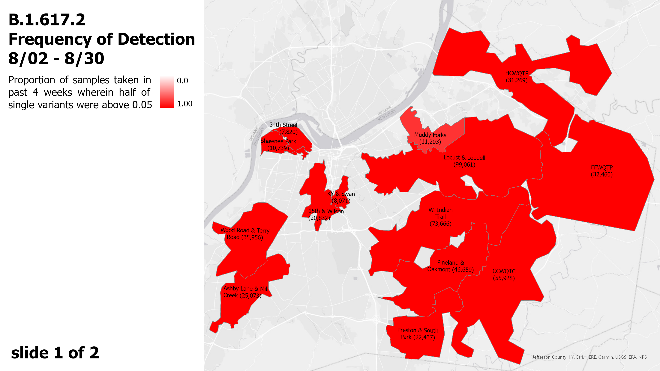

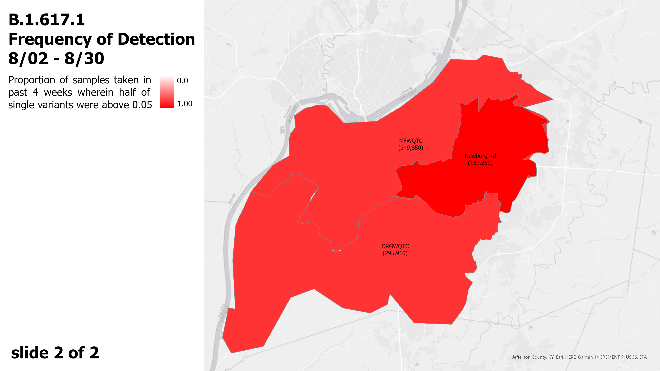


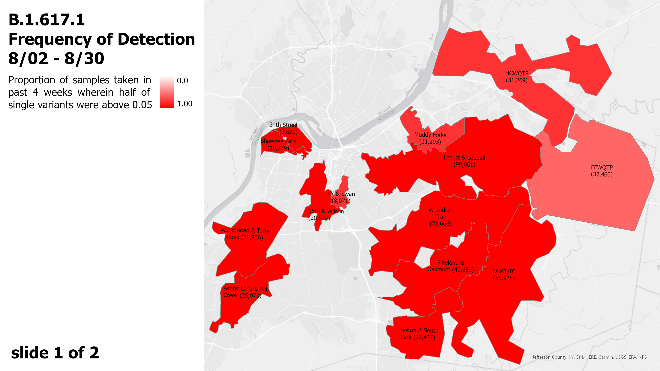

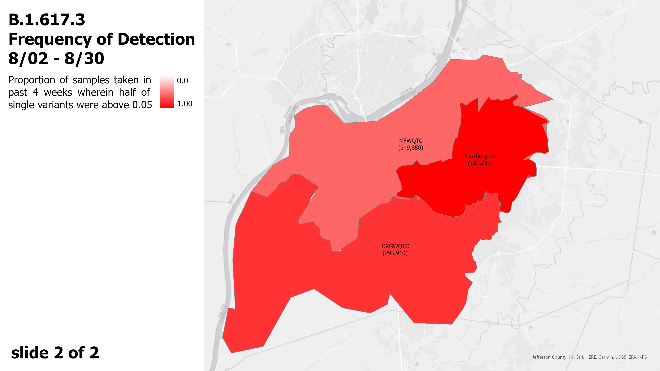


Fig S4 Example report from August 2021 of genomic surveillance of SARS-CoV-2 in wastewater data in Louisville/Jefferson County, Kentucky (USA), as presented weekly by academic partners to Louisville Metro Public Health and Wellness.
